# Assembly rules in a resource gradient: Competition and abiotic filtering determine the structuring of plant communities in stressful environments

**DOI:** 10.1371/journal.pone.0230097

**Published:** 2020-03-13

**Authors:** Bruno Sousa Menezes, Fernando Roberto Martins, Ellen Cristina Dantas Carvalho, Bruno Cruz Souza, Andrea Pereira Silveira, Maria Iracema Bezerra Loiola, Francisca Soares Araújo

**Affiliations:** 1 Department of Biology, Science Centre, Federal University of Ceará—UFC, Fortaleza, CE, Brazil; 2 Department of Plant Biology, Institute of Biology, University of Campinas—UNICAMP, Campinas, SP, Brazil; 3 Itapipoca Faculty of Education, Ceará State University—UECE, Itapipoca, CE, Brazil; Hainan University, CHINA

## Abstract

The relative importance of different community assembly mechanisms varies depending on the environment. According to the stress-dominance hypothesis (SDH), assembly mechanisms range from strong abiotic filtering to competition as the environment becomes more favourable. Most evidence for the SDH comes from studies in gradients of conditions (i.e. abiotic environmental factors that influence the functioning of organisms but are not consumed by them). However, we hypothesized that in resource gradients, competition increases as abiotic filtering becomes stronger. To test our hypothesis, we set up eight plots at different sites along an abiotic severity gradient in the Brazilian semi-arid region (BSAR). In each plot, we identified and measured each woody plant species found, and we recorded 11 functional traits of the main species, dividing the traits into alpha (competition effects) and beta (abiotic filtering effects). We investigated the presence of phylogenetic signal in the traits, the community phylogenetic and phenotypic patterns, and associated the variation in these patterns with the availability of water and soil nutrients. We found phylogenetic signal for most (91%) of the traits analysed. The phylogenetic patterns varied from clustered in stressful sites to random or overdispersed in favourable sites, and we concluded that these phylogenetic patterns were the result of historical processes influencing community assembly in different environments in the BSAR. In general, the phenotypic patterns varied from clustered at the most stressful end to random at less stressful sites. Our results show that in resource gradients, any restriction of the resource (hydric or edaphic) intensifies abiotic filtering and, at the same time, increases the competitive hierarchy among species. On the other hand, stochastic processes seem to have a stronger influence under more favourable abiotic conditions, where abiotic filtering and competition are weaker. Thus, we conclude that the SDH is not supported in resource gradients.

## Introduction

Community structure refers to the arrangement, order, and relationships among species that form a community [1]. Thus, to know the community structure is important because by it we can infer how species coexist sharing available resources in the environment. The way species are selected in the regional pool and fit together to coexist in local communities is defined by assembly rules [2]. Three different perspectives are often used to explain community assembly rules: historical, niche-related, and neutral [3].

From a historical perspective, species distributions can be determined largely by biogeographical processes that involve evolutionary forces and large-scale dispersal [3,4]. The evolutionary relatedness among lineages of the regional pool and the dispersal limitation can alter patterns of community assembly established through ecological sorting processes [3]. Thus, the biogeographic filters can prevent at least some species from reaching some sites. In turn, not all species that arrive at a site can withstand the site-specific abiotic factors: according to the niche-related perspective, the environment can act as a selective force (abiotic filtering mechanism), enabling only species with similar functional traits to establish under those abiotic factors [5]. Additionally, not every species that arrives and establishes at a site can persist as a population. According to niche-related biotic filtering, ecologically similar species tend to be excluded by interspecific competition [6]. Finally, the neutral perspective proposes an alternative to niche-related perspective, in which all species in a community are functionally equivalent and compete equally for resources [7]. So any species, functionally similar or not, that arrives at a site can establish and persist regardless of biotic and abiotic factors [7].

Since Webb *et al*. [8], community assembly mechanisms have often been inferred from phylogenetic patterns, seldom combined with phenotypic patterns and presence of phylogenetic signal. When there is phylogenetic conservativism, closely related species tend to have more similar functional traits than distantly related ones [9]. In this case, the abiotic filtering leads to clustering of both phylogenetic and phenotypic patterns [8]. If competition prevails, the phylogenetic and phenotypic patterns are both expected to be overdispersed [8]. However, in some cases when there is a competitive hierarchy, competition (asymmetric competition) can also generate clustered patterns even if the traits are conserved [10]. In asymmetric competition, competitively superior species tend to exclude inferior (functionally distinct species), that are phylogenetically more distant, due to phylogenetic conservatism [10]. On the other hand, when there is no phylogenetic conservatism, the interpretation of the patterns changes. Abiotic filtering generates overdispersion because species of distinct clades with similar traits (phenotypic attraction) are selected for the same environment and competition (symmetric or asymmetric) creates clustered or random patterns because the competitive exclusion of functionally similar species (phenotypic repulsion) may occur on a phylogenetically close clade or not [8]. When stochastic processes prevail on community structure, both the phylogenetic and phenotypic patterns should be random, independent of the evolutionary history of the traits, due to the species functional equivalence [11]. The balance between competition and abiotic filtering can also generate phylogenetic patterns indistinguishable from random, but in this case, the phenotypic patterns should indicate attraction or repulsion [11].

Most studies on community assembly have inferred the role of different mechanisms by taking into account only phylogenetic patterns, thus leading to inconsistent conclusions [12]. Due to the complexity involved in detecting community assembly mechanisms, Lopez *et al*. [13] recently proposed dividing phenotypic patterns into alpha- and beta-traits to represent competition effects and abiotic filtering, respectively. Separating alpha- and beta-traits in the analysis of phenotypic patterns, prevents competition and environmental filtering masking one another, and also reduces the likelihood that these two processes will be confused when unexpected or ambiguous patterns are generated [13]. When abiotic filtering is strong and competition is weak, alpha-trait patterns are always random, regardless of phylogenetic conservatism, because competition has little influence on the community; whereas beta-traits are expected to be clustered if there is phylogenetic conservatism, or overdispersed if there is no phylogenetic conservatism [13]. If competition is strong and abiotic filtering weak, alpha-traits should be overdispersed when there is phylogenetic conservatism, and clustered or random when there is no phylogenetic conservatism; whereas beta-traits are always random [13]. The phylogenetic patterns in each situation depend on the conservatism of the traits. If both beta- and alpha-traits and are conserved, phylogenetic patterns vary from clustered to overdispersed as environmental conditions become less stressful. If beta-traits are conserved, but alpha-traits are not, only the beta-traits will influence the phylogenetic patterns, which should follow the same variation as the beta-traits along the stress gradient (from clustered in stress situations to random in favourable situations). If alpha-traits are conserved, but beta-traits are not, the phylogenetic patterns will follow the pattern of the alpha-trait (vary from random in stress situations to overdispersed in favourable situations). If neither alpha- nor beta-traits are conserved, the phylogenetic patterns should be random, no matter how stressful the environment [13].

Abiotic filtering, competition, and neutral processes are thought to act together in community assembly, with the relative importance of each one of them varying as the environment varies. Weiher and Keddy [14] have stated that abiotic filtering predominates in stressful environments and weakens in favourable environments, while competition acts conversely: its role is weak in high stress situations but becomes increasingly important as the environment become more benign. This model, recently cited in the literature as the stress-dominance hypothesis–SDH [15], has been tested in different types of environmental gradients [15–26]. Studies have provided support for the SDH when considering the variation in the relative importance of the community assembly mechanisms in relation to gradients of abiotic conditions, such as decreasing temperature [17–20, 22, 26] or increasing fire intensity [16]. However, Coyle *et al*. [15] showed that the SDH did not explain the variation of the community assembly mechanisms in relation to resource gradients, such as availability of water and soil nutrients. Thus, it is possible that the SDH holds only for gradients of conditions (e.g. temperature, fire).

**Fig 1:**
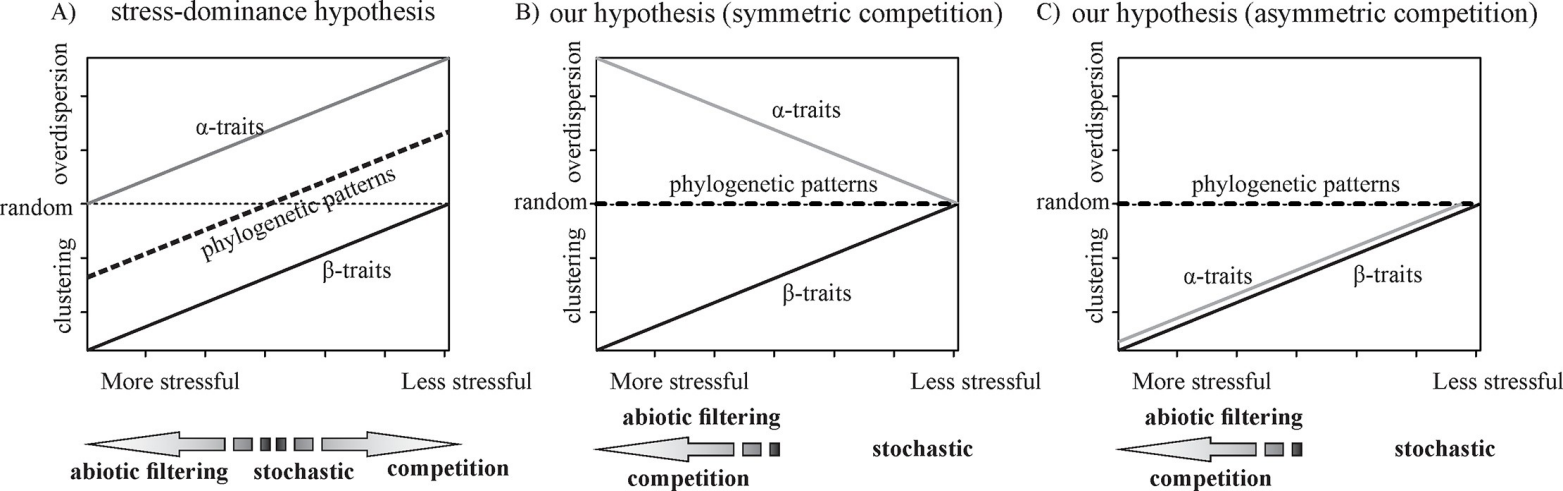
Hypothetical model of the variation in phylogenetic and phenotypic patterns along the abiotic gradients in limiting environments. A–Model expected by stress-dominance hypothesis, when the gradient is driven by an abiotic condition. B, C–Our proposed model, when the gradient is driven by a disputed resource (B–expectation for symmetric competition and C—expectation for asymmetric competition).

In this study, our goal was to investigate the variation in community assembly mechanisms across strong gradients of water and soil nutrient restriction, within the context of the SDH. As the type of gradient analysed is driven by resources instead of a condition, we hypothesize that competition can increase as abiotic filtering becomes stronger. To address this hypothesis, we assumed phylogenetic conservatism and investigated both phylogenetic and phenotypic (alpha- and beta-trait) patterns in tree communities across a gradient of water and soil nutrients. If the SDH holds, that is, if abiotic filtering increases and competition decreases across a gradient of increasing severity, we expect that: a) phylogenetic patterns range from clustered in stressful sites to overdispersed in less stressful sites; b) phenotypic patterns of beta-traits vary from clustered to random along the gradient; and c) phenotypic patterns of alpha-traits range from random in stressful sites to overdispersed in less stressful sites (Fig 1A). However, if both competition and abiotic filtering increase as the environment becomes more stressful, we expect that: a) phylogenetic patterns are random and do not vary along the gradient (in stressful sites the pattern is random due to balance between abiotic filtering and competition and in the more favourable sites the randomness is a consequence of stochastic processes); b) phenotypic patterns of beta-traits vary from clustered to random across the gradient; and c) phenotypic patterns of alpha-traits range from overdispersed (or clustered if the competition is asymmetric) in stressful sites to random in less stressful sites (Fig 1B and 1C).

## Materials and methods

### Study area

The Brazilian semi-arid region (BSAR) occupies 1,128,697 Km^2^ in northeastern Brazil [27]. The predominate climate is hot and dry, with high temperatures throughout the year (around 26ºC), which result in high annual potential evapotranspiration (1,500 to 2,000 mm.year^-1^) [28,29]. The high evapotranspiration together with low rainfall (between 500 and 750 mm.year^-1^; [29]) results in high water deficits. Rainfall is seasonal (concentrated in 3 to 4 months), and erratic, with an irregular distribution even during the rainy season [28]. The main geologic units in the region are the Proterozoic crystalline basement (most of the semiarid area) and the Paleozoic and Mesozoic sedimentary basins [29]. Soils on the crystalline basement tend to be shallow, clayey and rocky, usually classified (according to the Brazilian Soil Classification System) as lithic neosoils (leptosols in WRB/FAO; lithic endoaquents in USDA Soil Taxonomy), regolithic neosoils (regosols; psamments) and luvisols (luvisols; alfisols) [29]. Soils on sedimentary material tend to be deep and sandy, usually classified as latosols (ferralsols; oxisols), luvisols (luvisols; aridisols) and quartzarenic neosoils (arenosols; quartzipsamments) [29]. There are edapho-climatic gradients of humidity of several scales, which are generally associated with distance from the coast, soil depth, altitude, and relief dissection, slope and aspect (leeward or windward) [30]. The proximity of the coast and the increase in altitude reduce temperature and augment precipitation and consequently enhance water availability. The interactions between climate, distance to coast, soil, and relief generate very different environments leading to different vegetation formations. Considering Holdridge’s life zones [31,32], the BSAR is the most heterogeneous region in Brazil, with 24 life zones, of which 16 are ecotones [33]. The modal life zones are Basal Moist Tropical Forest, Premontane Moist Tropical Forest, Basal Moist Subtropical Forest, Basal Dry Tropical Forest, Premontane Dry Tropical Forest, Basal Dry Subtropical Forest, Basal Very Dry Tropical Forest, and Thorn Premontane Tropical Woodland [33]. Among these life zones, the Basal Very Dry Tropical Forest is predominant.

**Fig 2:**
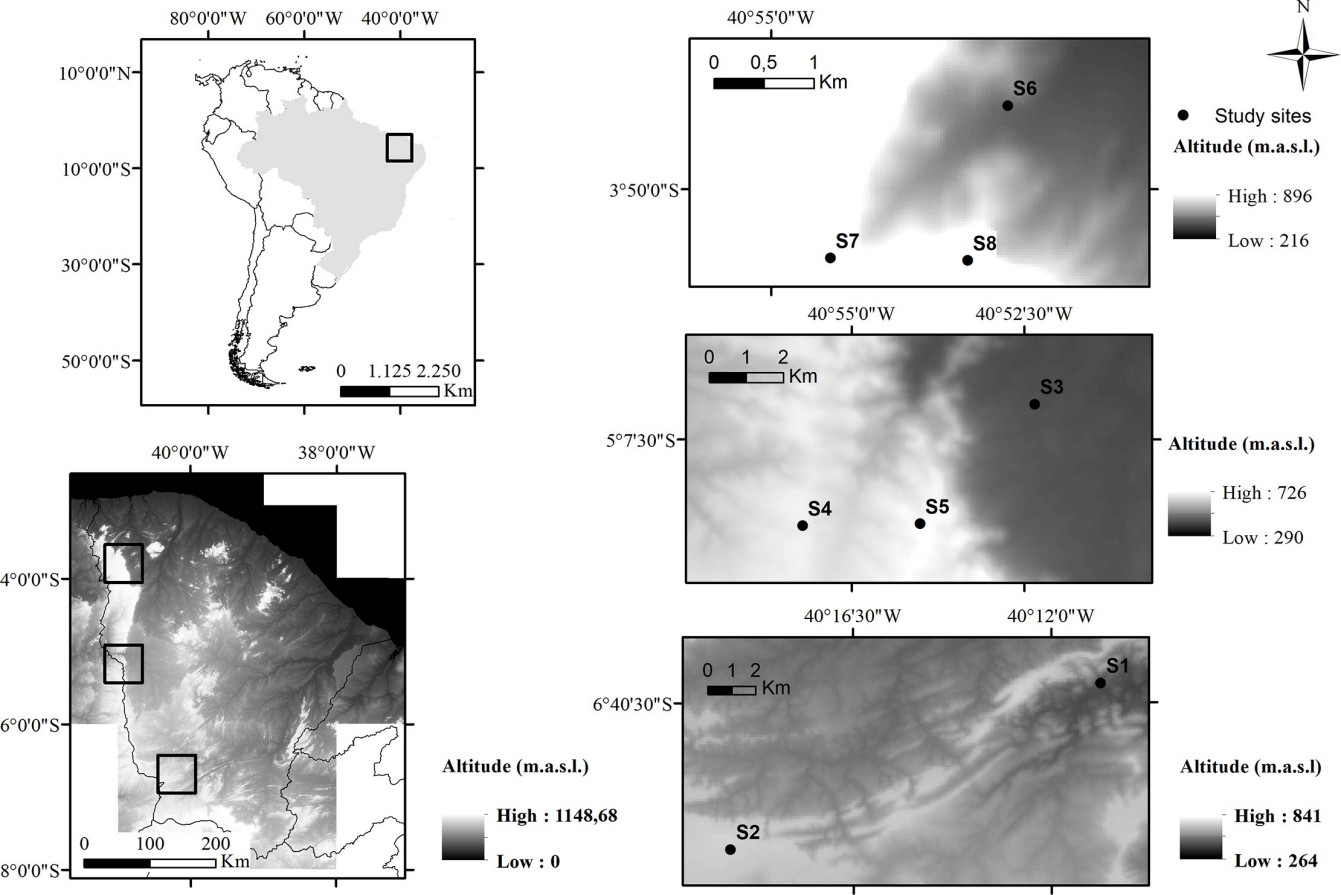
Digital elevation model of the *Meio Norte* sedimentary basin and adjacent areas (crystalline basement) with the location of study sites.

We analysed abiotic gradients in the Ibiapaba Plateau; the western *cuesta* of the *Meio Norte* sedimentary basin; and in adjacent areas of the crystalline basement complex, in the semi-arid region of northeastern Brazil (Fig 2). The *Meio Norte* sedimentary basin covers the entire western part of the state of Ceará, from north to south, through a continuous, abrupt, and quite scalloped escarpment [34]. It is an asymmetric cuesta formed by the lifting of the eastern border of the *Meio Norte* sedimentary basin, whose front (windward) is elevated to the east and contrasts with the flattened reverse (leeward) to the west, toward the Poti river, at the border of Piauí state. At the eastern front of the cuesta lies the flattened, highly eroded peripheral depression, constituted by the crystalline basement complex [34].

The crystalline basement complex represents the driest extreme of the gradient we analysed, with a BSh (Köppen-Geiger system) climate [35], average total annual rainfall of 450–900 mm, average total annual evapotranspiration about 1,500 mm, and altitudes between 300 and 450 m.a.s.l (Table 1). The predominant soils have a loam and sandy loam texture, are in general fertile, shallow and stony (Table 1, details in S1 Table). In the crystalline basement, the vegetation varies from Basal Very Dry Tropical Forest (300–450 m.a.s.l) to Basal Dry Tropical Forest (about 450 m.a.s.l). The Basal Very Dry Tropical Forest, locally called *caatinga*, is constituted by mostly spinescent and deciduous trees, forming only one woody layer 5–6 m high, with an open canopy and scattered emergent trees (Table 1). The Basal Dry Tropical Forest has deciduous trees that constitute two layers, a 6-m understory and a > 9-m closed canopy.

**Table 1:** Abiotic, physiognomic, and edaphic characteristics of different woody communities in the *Meio Norte* sedimentary basin and adjacent areas of the crystalline basement complex of the semi-arid region of northeastern Brazil.

Sites (cod)	Locality Municipality	Latitude Longitude	Substrate	Altitude (m asl)	Rainfall and Evapotranspiration (mm.year^-1^)	Plant formation	Density (ind.ha^-1^) and Basal area (m^2^.ha^-1^)	Soil Depth (cm)	Textural Class	Potential acidity (cmol_c_.kg^-1^)	pH	Available water content (g.kg^-1^)
S1	Aiuaba	-6.6678	crystalline	460	474	Basal Very Dry Tropical Forest	3,190	187	Loam	3.05	4.6	7.24
	Aiuaba, CE	-40.181			1,398		34.68					
S2	Monte Castelo	-6.7304	sedimentary	675	529	Premontane Very Dry Tropical Forest	3,656.7	+200	Clay loam and Sandy clay loam	4.95	4.2	5.91
	Aiuaba, CE	-40.321			1,230		16.65					
S3	Poti	-5.1162	crystalline	300	761	Basal Very Dry Tropical Forest	1,960	34	Sandy loam and Loamy sand	3.47	5.3	6.25
	Crateus, CE	-40.872			1,551		31.19					
S4	Buriti dos Montes	-5.1458	sedimentary	645	898	Premontane Dry Tropical Forest	6,096.7	+200	Sand and Loamy sand	3.96	4.6	1.40
	Buriti dos Montes, PI	-40.929			1,230		20.17					
S5	Tucuns	-5.1414	sedimentary	685	945	Premontane Dry Tropical Forest	5,860	+200	Loamy sand and Sandy loam	5.45	4.3	2.41
	Crateus, CE	-40.901			1,159		47.50					
S6	Araticum	-3.8258	crystalline	450	1,131	Basal Dry Tropical Forest	3,023.3	70	Sandy loam and Loam	7.51	5.3	7.62
					1,496		41.28					
	Ubajara, CE	-40.895										
S7	Ubajara	-3.8395	sedimentary	825	1,205	Premontane Moist Tropical Forest	1,173.3	+200	Sandy loam	5.61	4.5	7.70
	Ubajara, CE*	-40.911			1,029		28.47					
S8	Ubajara	-3.8397	sedimentary	845	1,383	Premontane Moist Tropical Forest	1,533.3	+200	Sandy loam	5.61	4.5	7.70
	Ubajara, CE	-40.899			1,017		39.10					

* Rainfall and temperature data of this site was collected from Jaburuna, Ubajara, CE because it was the closest locality to the sample unit with a similar altitude.

On the Ibiapaba cuesta, the climate is As (Köppen-Geiger system; [35]), with high rainfall (530–1,400 mm) and a lower temperature than the crystalline basement due to increased altitude. The altitude varies from 650 m to 850 m at the ridge. The soil, in general, is poor in nutrients, acid, highly leached, and has low water retention capacity (Table 1, details in S1 Table). From north to south, the vegetation varies from Premontante Moist Tropical Forest (850 m.a.s.l) to Premontane Dry Tropical Forest (about 700 m.a.s.l) on the windward side, and Premontane Dry Tropical Forest or Premontane Very Dry Tropical Forest (650–700 m.a.s.l) on the leeward side. Premontane Dry Tropical Forest has deciduous trees and two vegetation layers: a 5-m understory and a > 8-m closed canopy (Table 1). The Premontante Moist Tropical Forest, has non-spinescent trees constituting two woody layers: a 10-m understory and a >15-m canopy (Table 1). The vegetation on the leeward side of Ibiapaba is a Premontane Dry Tropical Forest or Premontane Very Dry Tropical Forest (depending on ratio between rainfall and evapotranspiration), locally called *carrasco*, and is composed of high density, 3–4 m tall, deciduous, non-spinescent shrub forming a single layer.

### Field sampling

We sampled eight sites covering altitudinal (from 300 m to 845 m a.s.l) and continentality (from north to south) gradients in the Ibiapaba Plateau and adjacent areas. The sites were located within three different conservation units (Aiuaba Ecological Station, Serra das Almas Natural Reserve, and Ubajara National Park) in order to minimize anthropogenic effects on the results (Fig 2). We set up 0.3-ha (30 m x 100 m) plots at each site. We identified all woody species present within these plots and recorded plant height and perimeter. In the Basal Very Dry Tropical Forest, Basal Dry Tropical Forest, Premontane Dry Tropical Forest, and Premontane Very Dry Tropical Forest we measured plants with a stem ≥ 9 cm at ground level (PGL ≥ 9 cm), because the trees had fine, multiple stems at ground level. In Premontane Moist Tropical Forest, we sampled plants with a perimeter ≥ 15 cm at breast height (PBH) because the trees were taller. We transformed perimeters into diameters to calculate individual biomass. We recorded plants with multiple stems as a single diameter by pooling all individual stems together.

We considered average annual temperature and average total annual rainfall as climate variables and soil physicochemical descriptors as soil variables. Climate variables were obtained from the *Climate-data*.*org* database from the closest locations to the study sites. We used these data to calculate the climatic water balance at the sites (S2 Table), following Thornthwaite and Mather [36]. In exceptional cases, when the annual value of the sum of rainfall (R)—potential evapotranspiration (PET) was negative (i.e., Σ[R-PET] < 0) and water holding capacity (WHC) was higher than the sum of positive values of R-PET (Σ[R-PET]^+^), we calculated the water balance from a simplified method suggested by Pereira [37]. We opened trenches and made the morphological description of soils at each site following Santos *et al*. [38]. We also collected samples in each horizon for physicochemical analyses at the Laboratory for Analysis of Soil, Water, and Fertilisers at the Federal University of Ceará.

We analysed granulometry data (sand, silt, and clay content), available water content, bulk density, pH, electric conductivity, soil adsorption complex (Ca^2+^, Mg^2+^, Na^+^, K^+^, Al^3+^), potential acidity (H^+^+Al^3+^), cation exchange capacity, sum of basic cations, assimilable phosphorus, nitrogen, carbon, and organic matter (S1 Table). We used the total dispersion method for granulometry. We obtained the available water content through the difference between water content at field capacity (0.033MPa) and permanent wilting point (1.5MPa), measured with a Richards extractor. We determined the cation exchange using Mehlich-1 extraction by K^+^, Na^+^, and P, KCl extraction by Ca^2+^, Mg^2+^, and Al^3+^, and ammonium acetate by potential acidity (H^+^+Al^3+^). We used the Kieldahl method for nitrogen determination. We carried out all soil physicochemical analyses following protocols established by Embrapa [39].

### Trait measurements

We recorded 11 functional traits (maximum height, mean biomass, specific leaf area, leaf size, leaf nitrogen concentration, leaf phenology, leaf type, wood density, potential hydraulic conductivity, dispersal mode, and dispersule size) of each species present at the study sites with at least five individuals (total = 142 species), following Cornelissen *et al*. [40] and Pérez-Harguindeguy *et al*. [41]. Species that occurred in more than one site were considered as different species. We excluded lianas and small shrubs due to the impossibility of collecting branches for the analysis of stem traits. We chose these traits because they are related to competitive ability and drought response (S3 Table).

We selected three individuals per species and collected five leaves and three branches per individual. We measured height with a 15.24 m telescopic pole and calculated the mean height of each species by sampling five individuals at random. We estimated the plant biomass of the Basal Very Dry Tropical Forest, Premontane Very Dry Tropical Forest, Basal Dry Tropical Forest, and Premontane Dry Tropical Forest using the equation of Sampaio and Silva [42] and it was calculated for the Premontane Moist Tropical Forest using the equation of Brown [43]. In both estimations, we considered the diameter as the independent variable. We did not include the petioles in the calculation of leaf area. For the stem traits, we used only the heartwood and sapwood of branch samples (approximately 3 cm), removing all bark. We determined the wood density by dividing the sample dry mass (dried for 72 h) by the volume calculated using the Archimedes principle [44]. We estimated the potential hydraulic conductivity from wood density data, using equations proposed by Martinez-Cabrera *et al*. [45]. For the leaf phenology, we divided the species into deciduous and evergreen. We defined deciduous species as those that spent at least one month without leaves. We also classified semideciduous species as deciduous due to the difficulty in differentiating between these two types. We classified the dispersal mode into anemochory, barochory, autochory, and zoochory, following Pijl [46] and dispersule size into small (< 0.6 cm in length), medium (0.6–1.5 cm), large (1.6–3.0 cm), and very large (> 3.0 cm), following Tabarelli and Peres [47]. We compiled leaf phenology, dispersal mode, and dispersule size data from literature and exsiccates deposited in the *Prisco Bezerra* Herbarium of the Federal University of Ceará.

### Phylogenetic and phenotypic trees

We constructed a phylogenetic tree including species from all sites (regional species pool) using Phylocom v. 4.2 software [48]. The taxa were arranged according to APG IV classification system, and the species nomenclature according to Brazilian Flora 2020. We confirmed all species names using the Plantminer tool [49]. We used the tree R20160415 [50] as a reference, dating the clades following Magallón and Castillo [51]. Undated nodes were estimated with the BLADJ (Branch Length Adjuster) algorithm. Within-family polytomies were resolved using published phylogenies: Anacardiaceae [52], Annonaceae [53], Euphorbiaceae [54], Fabaceae [55,56], Malvaceae [57], Myrtaceae [58], Rubiaceae [59], and Salicaceae [60].

We constructed a phenotypic tree with the species for which we had collected trait data only. We standardized trait values (average = 0; standard deviation = 1) because they were measured at different scales. We built a distance matrix, after transforming differences in species traits into functional distances, using the generalized Gower distance [61]. We made a UPGMA cluster analysis to build a functional dendrogram. Next, we transformed the dendrogram into a phylogenetic tree, so that phylogenetic and phenotypic patterns could be calculated based on the same metrics. We built a functional tree using the “picante” package in R [62].

### Data analyses

#### Abiotic gradient

As our research objective was to characterize the variation trend of phylogenetic and phenotypic patterns along a stress gradient, we first investigated the abiotic gradient. We performed a principal component analysis (PCA) for both sets of variables: climatic and edaphic. We used the climatic variables; rainfall (R), potential evapotranspiration (PET), actual evapotranspiration (AET), water deficit (DEF), and water excess (EXC). We used the edaphic variables; granulometry, soil available water content (AWC), bulk density (BD), pH, cation exchange capacity (CEC), sum of basic cations (S), electric conductivity (EC), potential acidity (H^+^+Al^3+^), organic matter (OM), and macro- and micronutrients (Ca, Mg, Na, K, Al, P, C, and N). In the PCA analyses, we used only non-collinear variables (Pearson’s r < 0.7). We assessed the importance of ordination axes through a comparison between the real variation represented by individual PCA axes and the relevant variation calculated by the broken-stick model [63]. We also tested the correlation between variables and the PCA axis to identify which variables were the most important for ordination. All analyses were carried out in the “vegan” package [64].

#### Phylogenetic signal

To infer the role of community mechanisms based on the phylogenetic and functional patterns, we first tested the phylogenetic signal of the functional traits of species in the regional pool. We carried out the analysis for each trait separately. For continuous traits, we identified the signal through the analysis of phylogenetic independent contrast–PIC [65]. If phylogenetically close species show more similar functional traits, the independent magnitude of contrast is similar across phylogenetic trees and therefore, the PIC value is low. We compared the observed contrast value to the expectations under a null model of randomly swapping trait values across the tips of the tree, with 999 randomizations. For categorical traits, we assessed the phylogenetic signal by comparing the minimum number of character state changes across the tree to a null model (999 randomizations) in which the trait states were randomized at the tips of the trees [66]. If related species are similar to each other, the number of character state changes will be lower than expected at random. We performed the analyses for continuous traits using the “phylo.signal” function in the “picante” package in R [62] and the categorical traits using “phylo.signal.disc” functions.

#### Phylogenetic and phenotypic patterns

By ‘*phylogenetic pattern*’, we mean the arrangement of the phylogenetic distances between the taxa constituting the community. The phylogenetic distances may or may not differ from random, meaning that the community does or does not have a phylogenetic structure, respectively. The phylogenetic structure may be overdispersed (phylogenetic repulsion) when the phylogenetic distances are longer than those expected at random, or clustered (phylogenetic attraction) when the distances are shorter than expected [8]. By ‘*phenotypic pattern*’, we mean the arrangement of the distances among the functional traits of the species constituting the community. The community phenotypic pattern may or may not have a structure, which can be overdispersed (phenotypic repulsion) or clustered (phenotypic attraction) if the morphological distances are longer or shorter than those expected at random, respectively [8].

Three steps are important to test the phylogenetic and functional patterns: 1) definition of the regional pool; 2) choice of distance metrics; and 3) construction of the appropriate null models. According to Pärtel *et al*. [67], regional pool refers to the set of species occurring in a region that are potentially capable of colonizing any local community. Thus, we defined that our regional pool would consist of all the species present in the eight sites. We realized phylobetadiversity analysis to validate the existence of a single regional pool among sites (S1 Text). Although the sites at the extremes of the gradient are separated by about 320 km, previous phylobetadiversity analyses showed a predominance of phylogenetic clustering among sites, i.e., species from different communities were phylogenetically closer than expected at random (data in S1 Text). In addition, the turnover index of phylobetadiversity showed weak relation with spatial distance (data in S1 Text). Hence, we can affirm that, despite some families being exclusive to more humid sites (S7 and S8), most clades occur at all sites. Thus, we had strong evidence to assume that all eight sites represent a single species pool. We used the mean pairwise distance–MPD as a phylogenetic distance measure [8] and the mean pairwise trait distance–PW (a metric that is similar to MPD but calculated with a phenotypic tree) as a functional distance measure. Because species richness influences these metrics, we standardised the values of the metrics through the standardised effect size (SES). The SES is the difference between observed and simulated values of the phylogenetic (or functional) metrics divided by the standard deviation of the simulated measures: SES = (obs.value–rnd.value) / sd.rnd.value. To make sure that the use of SES was correct, we tested normality and the asymmetry coefficient of null-distributions (S1 Fig). When these assumptions were not met, we made the SES correction, following Botta-Dukát [68]. To construct the null models, we used the *phylogeny*.*pool* algorithm. This algorithm creates random samples by drawing equally probable species from the regional pool and keeping the number of species equal to the original sample. Null models were constructed based on permutation tests with 999 simulations in each analysis. In these simulations, P-values ≤ 0.025 indicated a clustered structure, p-values ≥ 0.975 represented overdispersion, and values between 0.025 and 0.975 indicated randomness [8]. In addition, we divided phenotypic patterns into α-traits (maximum height, mean biomass, specific leaf area, leaf nitrogen concentration, dispersule size, leaf phenology, and potential conductivity), which are competition indicators, and β-traits (leaf size, wood density, dispersal mode, and leaf type), which are abiotic filtering indicators. We made this trait division based on the literature (see S3 Table). All phylogenetic and functional analyses were calculated using the “picante” package [62].

We tested for relationships between phylogenetic and functional SES values and stress gradients using multiple linear regression. Regarding models, we used significant PCA axes as independent variables and standardised MTD and PW values as dependent variables. We demonstrated the joint effect of axes on the metrics of phylogenetic and functional on a 3D scatterplot. Regression analysis were carried out in R and graphics made using the “scatterplot3d” package.

## Results

### Environmental gradient

**Fig 3:**
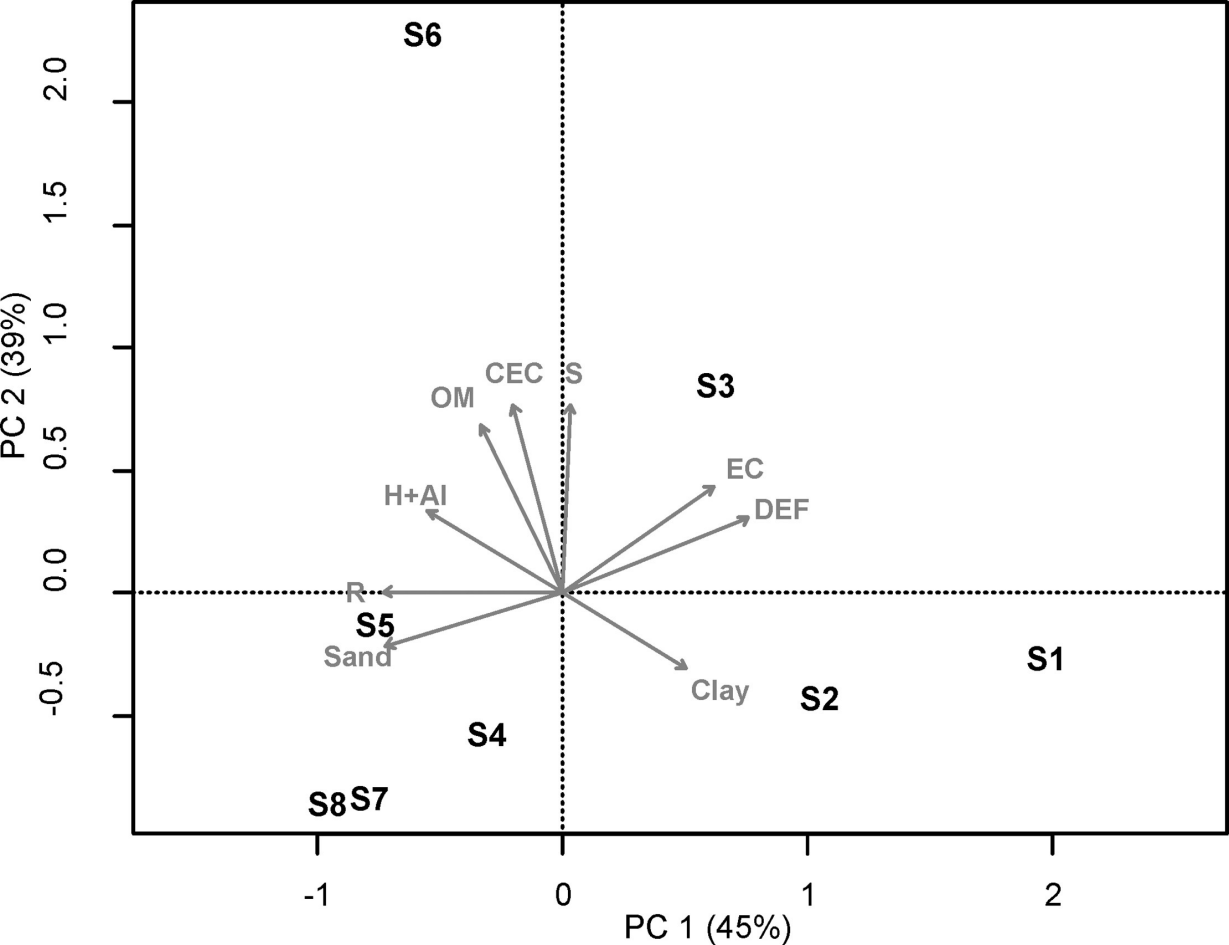
PCA biplot diagram ordering the subset of eight sites according to stress gradient. Site codes are presented in Table 1.

We found a stress gradient resulting from the interaction of two different resources: water and soil nutrient availability. Each set of abiotic variables was related to one of the two main axes of the PCA (Fig 3). The water availability gradient was described by the first principal component axis (PC1), which explained 45% of the variation among sites. Rainfall and water deficit showed high correlation with this axis (r = -0.45 and r = 0.42, respectively). For the soil nutrient gradient, the second principal component axis (PC2) explained an additional 39% of the data variation and was positively correlated with the sum of basic cations (S), cation exchange capacity (CEC), and organic matter (OM; r = 0.51, r = 0.51, and r = 0.45, respectively). In PCA analysis, only axes 1 and 2 showed eigenvalues larger than values expected at random (broken stick model, S4 Table). Thus, favourable sites in our gradient were characterized by high rainfall and fertile soils (site S6), while stressful sites were characterized by low rainfall and nutrient poor soil (sites S1 and S2). However, some sites with high rainfall were also stressful (sites S4 and S5), due to the nutrient restriction arising from sandy soil. The combination of high rainfall and sandy soil leads to high leaching, which results in acid soils, with low cation exchange capacity and basic cations.

### Phylogenetic signal

The observed PIC values for all the continuous traits (alpha and beta traits) analysed were lower than expected at random, indicating that close taxa share more similar trait values than those expected at random (phylogenetic signal; Table 2). This effect was significant in most traits (p < 0.025), except for leaf nitrogen concentration which was only slightly significant (p < 0.05). For all categorical traits (alpha and beta-traits), the minimum number of changes observed was lower than the number of changes at random, also confirming the presence of phylogenetic signal (Table 2). Thus, the fact that 91% of traits presented phylogenetic signal suggest there was phylogenetic conservatism.

**Table 2:** Phylogenetic signal of functional traits of woody species in abiotic gradients in the *Meio Norte* sedimentary basin and adjacent areas.

Traits	PICs obs	PICs rnd	Changes obs	Changes rnd	*p-value*
**alpha-traits**					
Hmax	2.29E-01	4.27E-01			**0.001**
Bmean	2.34E-04	4.55E-04			**0.002**
SLA	8.85E-01	13.39E-01			**0.002**
LNC	8.60E-01	11.03E-01			0.034*
Kp	2.17E+02	5.38E+02			**0.004**
LP			14	17	**0.006**
DS			35	54	**< 0.001**
**beta-traits**					
LS	6.17E+05	10.40E+05			**0.010**
WD	8.87E-05	17.50E-05			**0.001**
DM			18	49	**< 0.001**
LT			7	34	**< 0.001**

Phylogenetic signal calculated by phylogenetic independent contrast (PIC) for continuous traits: Hmax = maximum height (m); Bmean = mean biomass (Mg); SLA = specific leaf area (mm^2^.mg^-1^); LS = leaf size (mm^2^); LNC = leaf nitrogen concentration (mg.g^-1^); WD = wood density (mg.mm^-3^) and Kp = potential hydric conductivity (kg.m.MPa^-1^s^-1^); and by minimum number of changes for the categorical traits: DM = dispersal mode (anemochory, autochory, barochory, and zoochory), DS = dispersule size (small, medium, large, and very large), LT = leaf type (simple, compound, and bicompound), and LP = leaf phenology (deciduous or evergreen). Obs = observed; rnd = at random. Significant results (*p* < 0.025) are shown in **bold** (* = p < 0.05).

### Species richness

The regional species pool contained 174 species of 95 genera and 40 families. Community species richness varied from 19 to 62 species. The occurrence of some families was associated with decreased water restriction. For example, Malpighiaceae, Myrtaceae, Lauraceae, Melastomataceae, and Simaroubaceae were restricted to more humid areas, with the last three occurring only at the sites with the highest rainfall (S7 and S8). In contrast, Fabaceae, Boraginaceae, and Nyctaginaceae were less sensitive to the hydric gradient and occurred at all sites.

### Phylogenetic and phenotypic patterns

**Fig 4:**
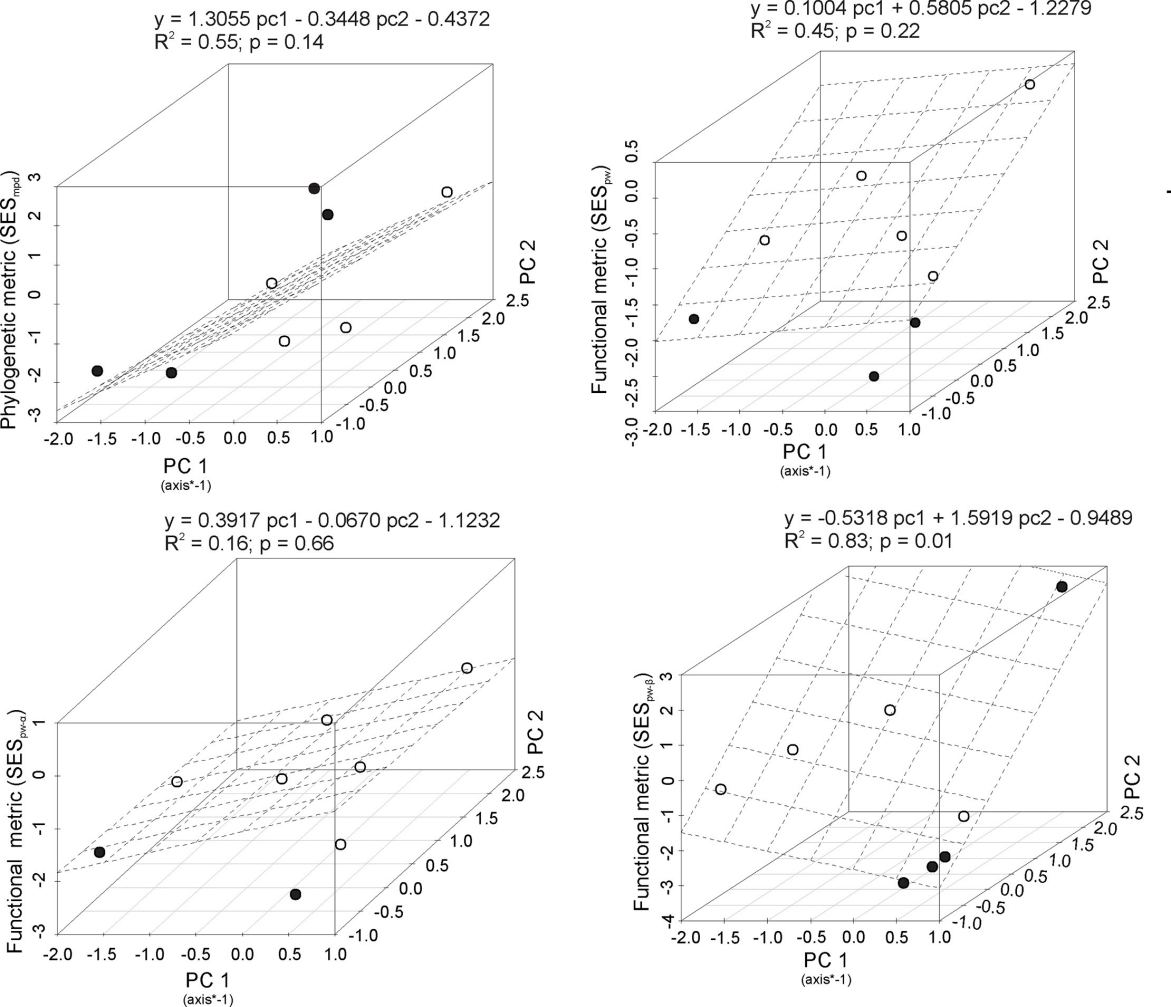
Variation in phylogenetic (SES_mpd_) and functional metrics (SES_pw_, SES_pw-α_, and SES_pw-β_) along stress gradients. Positive and significant values (*p-value* > 0.975) indicate overdispersion and negative and significant values (*p-value* < 0.025) indicate clustering. Significant values are represented by a solid circle.

The SES_mpd_ varied among communities from -2.35 to 2.80. Most communities showed a random pattern, but we found four communities in which results differed from expected at random: two with clustered and two with overdispersed patterns. Communities with a clustered structure occurred under higher abiotic restriction (low water and soil fertility) whereas those with an overdispersed structure occurred under conditions of high rainfall, but low soil nutrient availability. It was possible to observe a trend of changing of patterns from clustering to random along the stress gradient (Fig 4), although the sites with higher rainfall showed an overdispersed pattern.

For functional metrics, the variation in the beta-traits along a gradient was more evident than for general functional and alpha-traits (Fig 4). The β-trait patterns vary lineally from clustered to random along the gradient (Fig 4). We found the most negative values of SES_pw-β_ (-3.29; -2.61 and -2.31) in sites under high edaphic restriction, indicating strong abiotic filtering. In contrast, the only positive and significant value of SES_pw-β_ was recorded at the site with the lowest water and water restrictions, which confirmed the weak role of abiotic filtration under these conditions. For general and alpha traits, we did not find a significant relation along the gradient. However, sites under water, edaphic or both restrictions showed negative and significant (clustering pattern) for SES_pw_ and SESpw-α while sites without resource restriction showed values close to zero (random pattern; Fig 4). Thus, our results indicate that when there is a resource availability gradient, any resource restrictions should lead to clustered phenotypic structures.

## Discussion

The synthetic analysis of phylogenetic and phenotypic patterns of alpha and beta traits showed that competition and abiotic filtering acted together to structure the plant communities, and that their relative importance increased towards the same direction along the gradient. Environments with water or soil nutrient restrictions showed both strong abiotic filtering and limitation to similarity. As abiotic restriction decreased, the role of these mechanisms became weaker. This variation in the relative importance of the mechanisms along the gradient contradicts the stress-dominance hypothesis (SDH), in which abiotic filtering is stronger in stressful environments, whereas competition increases in more favourable environments [14,15]. This deviation is explained by the fact that the selection filters in our study were resources (water and soil nutrients) instead of conditions (e.g., fire or temperature), as is usually the case in studies which confirm the SDH (see [16–19, 21, 25]).

The interpretation of the phylogenetic pattern results is another issue that can influence the SDH confirmation. Most studies that support the SDH have used only phylogenetic data [18,20,21,25]. However, the mere detection of phylogenetic patterns is insufficient to elucidate the community assembly mechanisms [12,13]. Our study showed that, even with the presence of the phylogenetic signal, the phylogenetic and phenotypic patterns could vary along the gradient, independent of each other. This fact reinforces the argumentation of Cadotte et al. [69] that phylogenetic differences do not always predict ecological differences.

Due to the discrepancy between the phylogenetic and phenotypic pattern results, we decided to follow the recommendations of Gerhold *et al*. [12] and interpreted our phylogenetic patterns as evidence of historical processes instead of as a proxy for community assembly mechanisms. Thus, the phylogenetic clustering found in the most stressful extreme of our gradient probably resulted from historical processes related to the formation of the species pool. These high stress environments are located in the crystalline basement that was formed from modern pediplanation events that occurred during the Upper Tertiary [70]. Hence, the phylogenetic clustering should be a consequence of this recent evolutionary history. Additionally, the monophyletism and limited dispersion of many clades from seasonally dry tropical formations [71] may have also contributed to an increase in phylogenetic clustering.

The role of abiotic filtering in our study was inferred only based on the phenotypic patterns of beta-traits along the gradient. The occurrence of clustering patterns in sites with lower precipitation or base sum indicated that when there are water or edaphic limitations abiotic filtering is more intense. The performance of abiotic filtering along the analysed stress gradient was complex due to the presence of opposite abiotic gradients. The Ibiapaba cuesta has sandy and infertile soils due to higher leaching [29], so even in humid sites of our gradient, abiotic filtration can occur if the soil is nutrient poor. Without this clarity about the interaction of gradients it would be difficult to infer how mechanisms explained the community assembly in our study. Thus, in community structuring, studies along gradients are fundamental to evaluate several abiotic factors at the same time [72], especially in situations of opposite abiotic gradients. Analysis focused on a single main gradient [e.g. 15–26] and obscurity about the peculiarities of the gradient analysed can influence the SDH confirmation. Hence, the SDH should be confirmed in gradients in which both the phylogenetic structure of communities, and also the phenotypic patterns are assessed under the understanding that gradients interact with each other, e.g. water versus soil nutrient availability gradients.

The variation of alpha-trait patterns between stressful sites and more benign sites indicated that, when the gradient is formed by resources, and competition and abiotic filtering act in the same direction, competitive hierarchy explains the clustering in functional traits. According to Mayfield and Levine [10], when there is a difference in the competitive ability and similarities in niche preferences, superior competitors exclude less-fit taxa, generating a phenotypic clustering, even when traits are conserved. This perception about the effect of competition asymmetry was only possible due to the division of traits into alpha and beta, as suggested by Lopez *et al*. [13].

The randomness in the alpha and beta-trait patterns indicated a decrease in competition and abiotic filtering in the most favourable sites of the gradient. Additionally, higher water availability enables higher species richness and the occurrence of more distinct clades. Lauraceae and Burseraceae are examples of groups whose occurrence is associated with humid environments [73]. In our gradient, sites with rainfall greater than 900 mm should have a predominantly stochastic assembly, as predicted by the Neutral Theory by Hubbell [7]. The species occurrence should be limited by their dispersal capacity [74], and all taxa from the regional pool that arrive at the site should have the same probability of establishing themselves, regardless of their ecological characteristics.

Finally, the fact that we analysed only eight sites can be considered a limitation for our inferences. The absence of significant relationships along the gradient may be due to the low number of points analysed. However, the fact that we have analysed all possible combinations between the variation in the availability of water and soil nutrients gives us confidence to suppose that the introduction of a larger number of points will only confirm the trends found in our study. Thus, we conclude that in stress gradients formed by resource availability, greater water or edaphic restrictions lead to filtering of specific clades, low numbers of coexisting taxa, phenotypic clustering of beta- and alpha-traits due to the abiotic filtering and competitive hierarchy, respectively. At the humid extreme of the gradient, both competition for resources and abiotic filtering have lower intensity, stochastic processes should prevail which generates phenotypic randomness of alpha and beta-traits. Our study indicated that the SDH should not be corroborated in resource gradients.

## Supporting information

S1 Table Median physical and chemical soil characteristics at different sites in the *Meio Norte* sedimentary basin and adjacent areas of the crystalline basement complex. (DOC)

S2 Table Climatological water balance (Thornthwaite and Mather, 1955) at eight sites in the *Meio Norte* sedimentary basin and adjacent areas, semi-arid region of northeastern Brazil, with WHC = 100 mm (period 1982 to 2012). (T = temperature; R = rainfall; PET = potential evapotranspiration; NEG = negative accumulated; GW = ground water; ALT = GWactual–GWprevious; AET = actual evapotranspiration; DEF = water deficit; EXC = water excess).
(DOC)

S3 Table Traits used to measure the phenotypic pattern of woody species in different plant formations in the semi-arid region of northeastern Brazil. (DOC)

S4 Table Results of the comparisons between real variation represented by individual PCA axes and relevant variation calculated by the broken-stick model. (DOC)

S1 Fig Distributions of null model values for each metric analysed. (DOC)

S1 Text Phylobetadiversity analyses. (DOC)
