# Supplementary material for: Assembly rules in a resource gradient: Competition and abiotic filtering determine the structuring of plant communities in stressful environments
Source: PLoS One. 2020 Mar 13;15(3):e0230097. doi: 10.1371/journal.pone.0230097 (PMC7069682; doi:10.1371/journal.pone.0230097)
Supplement: S1 Table — (DOC) [file pone.0230097.s001.doc]

**S1 Table** – Median physical and chemical soil characteristics at different sites in the Meio Norte sedimentary basin and adjacent areas of the crystalline basement complex.

| **Characteristic** | **Sites** | | | | | | |
| --- | --- | --- | --- | --- | --- | --- | --- |
|  | S1 | S2 | S3 | S4 | S5 | S6 | S8 |
| Coarse sand, g.kg^-1^ | 188.00 | 118.00 | 399.50 | 310.50 | 98.00 | 105.00 | 14.00 |
| Fine sand, g.kg^-1^ | 161.50 | 291.00 | 319.50 | 582.00 | 699.00 | 412.00 | 576.00 |
| Silt, g.kg^-1^ | 424.50 | 305.00 | 195.50 | 44.50 | 126.00 | 388.50 | 245.00 |
| Clay, g.kg^-1^ | 227.00 | 271.00 | 93.50 | 76.50 | 101.00 | 95.00 | 153.00 |
| Clay dispersed by water, g.kg^-1^ | 7.50 | 133.00 | 38.50 | 11.50 | 42.00 | 59.50 | 126.00 |
| Bulk density, kg m^-^³ | 1.37 | 1.29 | 1.59 | 1.57 | 1.39 | 1.31 | 1.31 |
| Available water content (awc), g/100g | 7.24 | 5.91 | 6.25 | 1.40 | 2.41 | 7.62 | 7.70 |
| pH in water | 4.6 | 4.2 | 5.3 | 4.6 | 4.3 | 5.3 | 4.5 |
| Electric conductivity (EC), dS/m | 0.20 | 0.13 | 0.12 | 0.07 | 0.09 | 0.16 | 0.05 |
| Available Ca^b^, cmol_c_ kg^-^¹ | 0.70 | 0.20 | 3.85 | 0.65 | 0.50 | 5.50 | 0.10 |
| Available Mg^b^, cmol_c_ kg^-^¹^a^ | 0.65 | 0.20 | 2.05 | 0.60 | 0.50 | 1.65 | 0.20 |
| Available Na^a^, cmol_c_ kg^-^¹ | 0.09 | 0.08 | 0.03 | 0.02 | 0.02 | 0.52 | 0.08 |
| Available K^a^,  cmol_c_ kg^-^¹ | 0.36 | 0.08 | 0.31 | 0.02 | 0.05 | 0.31 | 0.03 |
| Available Al^b^, cmol_c_ kg^-^¹ | 1.85 | 3.05 | 0.25 | 0.88 | 1.30 | 3.48 | 3.30 |
| Potential acidity ^c^ (H^+^+Al^+3^), cmol_c_ kg^-1^ | 3.06 | 4.95 | 3.47 | 3.97 | 5.45 | 7.51 | 5.61 |
| Sum of basic cations (S), cmol_c_ kg^-1^ | 1.7 | 0.6 | 6.4 | 1.3 | 1.1 | 8.0 | 0.4 |
| Cation exchange capacity (CEC), cmol_c_ kg^-1^ | 4.7 | 5.5 | 9.9 | 5.1 | 7.1 | 15.5 | 5.9 |
| Assimilable phosphorus, mg.kg^-1^ | 4 | 1 | 1 | 8 | 10 | 14 | 1 |
| Nitrogen, g.kg^-1^ | 0.11 | 0.40 | 0.58 | 0.46 | 0.80 | 1.47 | 0.31 |
| Carbon, g.kg^-1^ | 1.17 | 4.27 | 6.09 | 4.35 | 8.22 | 13.53 | 3.25 |
| Organic matter, g.kg^-1^ | 2.02 | 7.36 | 10.50 | 7.50 | 14.17 | 23.32 | 5.60 |

Notes: Extracted using: aMehlich-1, b1 M KCl, cAmmonium acetate
