# Supplementary material for: Assembly rules in a resource gradient: Competition and abiotic filtering determine the structuring of plant communities in stressful environments
Source: PLoS One. 2020 Mar 13;15(3):e0230097. doi: 10.1371/journal.pone.0230097 (PMC7069682; doi:10.1371/journal.pone.0230097)
Supplement: S2 Table — (T = temperature; R = rainfall; PET = potential evapotranspiration; NEG = negative accumulated; GW = ground water; ALT = GWactual–GWprevious; AET = actual evapotranspiration; DEF = water deficit; EXC = water excess). (DOC) [file pone.0230097.s002.doc]

**S2 Table –** Climatological water balance (Thornthwaite and Mather, 1955) at eight sites in the Meio Norte sedimentary basin and adjacent areas, semi-arid region of northeastern Brazil, with WHC = 100 mm (period 1982 to 2012). (T = temperature; R = rainfall; PET = potential evapotranspiration; NEG = negative accumulated; GW = ground water; ALT = GWactual – GWprevious; AET = actual evapotranspiration; DEF = water deficit; EXC = water excess).

| **Months** | **T** | **R** | **PET** | **R-PET** | **NEG** | **GW** | **ALT** | **AET** | **DEF** | **EXC** |
| --- | --- | --- | --- | --- | --- | --- | --- | --- | --- | --- |
|  | **(ºC)** | **(mm)** | **(mm)** | **(mm)** |  | **(mm)** | **(mm)** | **(mm)** | **(mm)** | **(mm)** |
|  |  |  |  |  |  |  |  |  |  |  |
| *Site 1 - Aiuaba, CE* | | | | | | | | | | |
| Jan | 26.0 | 55 | 136.3 | -81.3 | -1,101.8 | 0.0 | 0.0 | 55.0 | 81.3 | 0.0 |
| Feb | 25.2 | 96 | 109.8 | -13.8 | -1115.6 | 0.0 | 0.0 | 96.0 | 13.8 | 0.0 |
| Mar | 24.5 | 127 | 109.4 | 17.6 | -173.6 | 17.6 | 17.6 | 109.4 | 0.0 | 0.0 |
| Apr | 24.3 | 99 | 101.4 | -2.4 | -176.0 | 17.2 | -0.4 | 99.4 | 2.0 | 0.0 |
| May | 23.9 | 31 | 98.1 | -67.1 | -243.1 | 8.8 | -8.4 | 39.4 | 58.7 | 0.0 |
| Jun | 23.7 | 9 | 91.7 | -82.7 | -325.8 | 3.8 | -4.9 | 13.9 | 77.7 | 0.0 |
| Jul | 23.9 | 5 | 97.7 | -92.7 | -418.5 | 1.5 | -2.3 | 7.3 | 90.4 | 0.0 |
| Aug | 24.7 | 3 | 110.0 | -107.0 | -525.6 | 0.5 | -1.0 | 4.0 | 106.0 | 0.0 |
| Sep | 25.6 | 3 | 121.4 | -118.4 | -644.0 | 0.2 | -0.4 | 3.4 | 118.0 | 0.0 |
| Oct | 26.2 | 5 | 137.3 | -132.3 | -776.3 | 0.0 | -0.1 | 5.1 | 132.2 | 0.0 |
| Nov | 26.5 | 12 | 141.3 | -129.3 | -905.6 | 0.0 | 0.0 | 12.0 | 129.3 | 0.0 |
| Dec | 26.4 | 29 | 143.9 | -114.9 | -1020.5 | 0.0 | 0.0 | 29.0 | 114.9 | 0.0 |
| Ann | 25.1 | 474.0 | 1,398.4 | -924.4 |  | 49.7 | 0.0 | 474.0 | 924.4 | 0.0 |
|  |  |  |  |  |  |  |  |  |  |  |
| *Site 2 – Monte Castelo, CE* | | | | | | | | | | |
| Jan | 24.8 | 67 | 120.0 | -53.0 | -808.6 | 0.0 | 0.0 | 67.0 | 52.9 | 0.0 |
| Feb | 23.9 | 106 | 96.1 | 9.9 | -230.6 | 10.0 | 9.9 | 96.1 | 0.0 | 0.0 |
| Mar | 23.3 | 136 | 97.4 | 38.6 | -72.1 | 48.6 | 38.6 | 97.4 | 0.0 | 0.0 |
| Apr | 23.1 | 108 | 90.4 | 17.6 | -41.2 | 66.2 | 17.6 | 90.4 | 0.0 | 0.0 |
| May | 22.7 | 31 | 87.7 | -56.7 | -97.9 | 37.6 | -28.7 | 59.7 | 28.1 | 0.0 |
| Jun | 22.5 | 9 | 82.1 | -73.1 | -171.0 | 18.1 | -19.5 | 28.5 | 53.6 | 0.0 |
| Jul | 22.7 | 5 | 87.4 | -82.4 | -253.4 | 7.9 | -10.1 | 15.1 | 72.2 | 0.0 |
| Aug | 23.3 | 3 | 95.4 | -92.4 | -345.8 | 3.2 | -4.8 | 7.8 | 87.6 | 0.0 |
| Sep | 24.3 | 3 | 105.9 | -102.9 | -448.6 | 1.1 | -2.0 | 5.0 | 100.8 | 0.0 |
| Oct | 25.0 | 7 | 120.6 | -113.6 | -562.3 | 0.4 | -0.8 | 7.8 | 112.9 | 0.0 |
| Nov | 25.3 | 20 | 122.6 | -102.6 | -664.9 | 0.1 | -0.2 | 20.2 | 102.4 | 0.0 |
| Dec | 25.1 | 34 | 124.8 | -90.8 | -755.6 | 0.1 | -0.1 | 34.1 | 90.7 | 0.0 |
| Ann | 23.8 | 529.0 | 1,230.2 | -701.2 |  | 193.2 | 0.0 | 529.0 | 701.2 | 0.0 |
|  |  |  |  |  |  |  |  |  |  |  |
| *Site 3 – Poti, CE* | | | | | | | | | | |
| Jan | 26.5 | 76 | 145.6 | -69.6 | -973.2 | 0.0 | 0.0 | 76.0 | 69.6 | 0.0 |
| Feb | 25.9 | 122 | 117.8 | 4.2 | -315.7 | 4.3 | 4.2 | 117.8 | 0.0 | 0.0 |
| Mar | 25.3 | 217 | 118.5 | 98.5 | 0.0 | 100.0 | 95.7 | 118.5 | 0.0 | 2.7 |
| Apr | 25.1 | 191 | 110.3 | 80.7 | 0.0 | 100.0 | 0.0 | 110.3 | 0.0 | 80.7 |
| May | 25.1 | 73 | 112.8 | -39.8 | -39.8 | 67.1 | -32.9 | 105.9 | 7.0 | 0.0 |
| Jun | 25.1 | 18 | 108.7 | -90.7 | -130.6 | 27.1 | -40.0 | 58.0 | 50.7 | 0.0 |
| Jul | 25.1 | 9 | 112.5 | -103.5 | -234.1 | 9.6 | -17.5 | 26.5 | 86.1 | 0.0 |

**S2 Table –** cont

| **Months** | **T** | **R** | **PET** | **R-PET** | **NEG** | **GW** | **ALT** | **AET** | **DEF** | **EXC** |
| --- | --- | --- | --- | --- | --- | --- | --- | --- | --- | --- |
|  | **(ºC)** | **(mm)** | **(mm)** | **(mm)** |  | **(mm)** | **(mm)** | **(mm)** | **(mm)** | **(mm)** |
| Aug | 26.3 | 2 | 134.1 | -132.1 | -366.2 | 2.6 | -7.1 | 9.1 | 125.1 | 0.0 |
| Sep | 27.1 | 1 | 143.0 | -142.0 | -508.2 | 0.6 | -1.9 | 2.9 | 140.1 | 0.0 |
| Oct | 27.3 | 3 | 151.3 | -148.3 | -656.6 | 0.1 | -0.5 | 3.5 | 147.9 | 0.0 |
| Nov | 27.3 | 11 | 148.0 | -137.0 | -793.6 | 0.0 | -0.1 | 11.1 | 136.9 | 0.0 |
| Dec | 26.7 | 38 | 148.0 | -110.0 | -903.6 | 0.0 | 0.0 | 38.0 | 110.0 | 0.0 |
| Ann | 26.1 | 761.0 | 1,550.8 | -789.8 |  | 311.5 | 0.0 | 677.6 | 873.2 | 83.4 |
|  |  |  |  |  |  |  |  |  |  |  |
| *Site 4 – Buriti dos Montes, PI* | | | | | | | | | | |
| Jan | 24.3 | 111 | 112.1 | -1.1 | -642.7 | 0.2 | 0.0 | 111.0 | 1.1 | 0.0 |
| Feb | 23.8 | 141 | 94.4 | 46.6 | -76.1 | 46.7 | 46.6 | 94.4 | 0.0 | 0.0 |
| Mar | 23.1 | 245 | 94.7 | 150.3 | 0.0 | 100.0 | 53.3 | 94.7 | 0.0 | 97.0 |
| Apr | 23.1 | 204 | 90.7 | 113.3 | 0.0 | 100.0 | 0.0 | 90.7 | 0.0 | 113.3 |
| May | 23.0 | 73 | 91.6 | -18.6 | -18.6 | 83.0 | -17.0 | 90.0 | 1.6 | 0.0 |
| Jun | 22.7 | 22 | 84.9 | -62.9 | -81.5 | 44.3 | -38.8 | 60.8 | 24.1 | 0.0 |
| Jul | 22.9 | 11 | 90.2 | -79.2 | -160.7 | 20.1 | -24.2 | 35.2 | 55.0 | 0.0 |
| Aug | 23.7 | 6 | 100.6 | -94.6 | -255.3 | 7.8 | -12.3 | 18.3 | 82.3 | 0.0 |
| Sep | 24.7 | 4 | 111.1 | -107.1 | -362.3 | 2.7 | -5.1 | 9.1 | 102.0 | 0.0 |
| Oct | 25.1 | 6 | 121.7 | -115.7 | -478.1 | 0.8 | -1.8 | 7.8 | 113.9 | 0.0 |
| Nov | 25.2 | 22 | 120.5 | -98.5 | -576.5 | 0.3 | -0.5 | 22.5 | 97.9 | 0.0 |
| Dec | 24.7 | 53 | 118.0 | -65.0 | -641.5 | 0.2 | -0.1 | 53.1 | 64.9 | 0.0 |
| Ann | 23.9 | 898.0 | 1,230.5 | -332.5 |  | 406.0 | 0.0 | 687.6 | 542.8 | 210.4 |
|  |  |  |  |  |  |  |  |  |  |  |
| *Site 5 – Tucuns, CE* | | | | | | | | | | |
| Jan | 23.8 | 102 | 107.3 | -5.3 | -575.0 | 0.3 | 0.0 | 102.0 | 5.3 | 0.0 |
| Feb | 23.1 | 149 | 88.4 | 60.6 | -49.6 | 60.9 | 60.6 | 88.4 | 0.0 | 0.0 |
| Mar | 22.5 | 253 | 90.0 | 163.0 | 0.0 | 100.0 | 39.1 | 90.0 | 0.0 | 123.9 |
| Apr | 22.3 | 215 | 84.0 | 131.0 | 0.0 | 100.0 | 0.0 | 84.0 | 0.0 | 131.0 |
| May | 22.3 | 92 | 85.9 | 6.1 | 0.0 | 100.0 | 0.0 | 85.9 | 0.0 | 6.1 |
| Jun | 22.2 | 33 | 81.8 | -48.8 | -48.8 | 61.4 | -38.6 | 71.6 | 10.2 | 0.0 |
| Jul | 22.4 | 17 | 86.8 | -69.8 | -118.6 | 30.5 | -30.8 | 47.8 | 39.0 | 0.0 |
| Aug | 23.1 | 8 | 95.3 | -87.3 | -205.9 | 12.8 | -17.8 | 25.8 | 69.6 | 0.0 |
| Sep | 24.0 | 6 | 103.7 | -97.7 | -303.7 | 4.8 | -8.0 | 14.0 | 89.8 | 0.0 |
| Oct | 24.4 | 6 | 113.5 | -107.5 | -411.2 | 1.6 | -3.2 | 9.2 | 104.3 | 0.0 |
| Nov | 24.5 | 18 | 112.3 | -94.3 | -505.5 | 0.6 | -1.0 | 19.0 | 93.3 | 0.0 |
| Dec | 24.0 | 46 | 110.2 | -64.2 | -569.6 | 0.3 | -0.3 | 46.3 | 63.9 | 0.0 |
| Ann | 23.2 | 945.0 | 1,159.3 | -214.3 |  | 473.3 | 0.0 | 684.0 | 475.3 | 261.0 |
|  |  |  |  |  |  |  |  |  |  |  |
| *Site 6 – Araticum, CE* | | | | | | | | | | |
| Jan | 26.3 | 115 | 138.9 | -23.9 | -825.5 | 0.0 | 0.0 | 115.0 | 23.9 | 0.0 |
| Feb | 25.7 | 184 | 115.0 | 69.0 | -37.1 | 69.0 | 69.0 | 115.0 | 0.0 | 0.0 |
| Mar | 24.8 | 297 | 111.7 | 185.3 | 0.0 | 100.0 | 31.0 | 111.7 | 0.0 | 154.4 |
| Apr | 24.9 | 277 | 108.6 | 168.4 | 0.0 | 100.0 | 0.0 | 108.6 | 0.0 | 168.4 |

**S2 Table –** conclusion

| **Months** | **T** | **R** | **PET** | **R-PET** | **NEG** | **GW** | **ALT** | **AET** | **DEF** | **EXC** |
| --- | --- | --- | --- | --- | --- | --- | --- | --- | --- | --- |
|  | **(ºC)** | **(mm)** | **(mm)** | **(mm)** |  | **(mm)** | **(mm)** | **(mm)** | **(mm)** | **(mm)** |
| May | 24.7 | 146 | 108.5 | 37.5 | 0.0 | 100.0 | 0.0 | 108.5 | 0.0 | 37.5 |
| Jun | 25.0 | 39 | 109.0 | -70.0 | -70.0 | 49.7 | -50.3 | 89.3 | 19.7 | 0.0 |
| Jul | 25.1 | 20 | 114.4 | -94.4 | -164.4 | 19.3 | -30.3 | 50.3 | 64.0 | 0.0 |
| Aug | 25.6 | 2 | 123.1 | -121.1 | -285.4 | 5.8 | -13.6 | 15.6 | 107.5 | 0.0 |
| Sep | 26.4 | 1 | 133.6 | -132.6 | -418.0 | 1.5 | -4.2 | 5.2 | 128.4 | 0.0 |
| Oct | 26.7 | 3 | 145.4 | -142.4 | -560.4 | 0.4 | -1.2 | 4.2 | 141.3 | 0.0 |
| Nov | 26.7 | 8 | 141.9 | -133.9 | -694.3 | 0.1 | -0.3 | 8.3 | 133.6 | 0.0 |
| Dec | 26.6 | 39 | 146.2 | -107.2 | -801.5 | 0.0 | -0.1 | 39.1 | 107.1 | 0.0 |
| Ann | 25.7 | 1,131 | 1,496.2 | -365.2 |  | 445.8 | 0.0 | 770.7 | 725.5 | 360.3 |
|  |  |  |  |  |  |  |  |  |  |  |
| *Site 7 – Jaburuna, CE* | | | | | | | | | | |
| Jan | 22.4 | 118 | 94.9 | 23.1 | -142.6 | 24.0 | 23.1 | 94.9 | 0.0 | 0.0 |
| Feb | 21.8 | 194 | 79.5 | 114.5 | 0.0 | 100.0 | 76.0 | 79.5 | 0.0 | 38.5 |
| Mar | 20.9 | 289 | 78.7 | 210.3 | 0.0 | 100.0 | 0.0 | 78.7 | 0.0 | 210.3 |
| Apr | 21.0 | 283 | 76.4 | 206.6 | 0.0 | 100.0 | 0.0 | 76.4 | 0.0 | 206.6 |
| May | 20.9 | 170 | 77.6 | 92.4 | 0.0 | 100.0 | 0.0 | 77.6 | 0.0 | 92.4 |
| Jun | 21.0 | 51 | 75.6 | -24.6 | -24.6 | 78.2 | -21.8 | 72.8 | 2.8 | 0.0 |
| Jul | 21.2 | 22 | 80.1 | -58.1 | -82.7 | 43.7 | -34.5 | 56.5 | 23.7 | 0.0 |
| Aug | 21.8 | 7 | 86.3 | -79.3 | -162.0 | 19.8 | -23.9 | 30.9 | 55.3 | 0.0 |
| Sep | 22.3 | 6 | 89.0 | -83.0 | -245.0 | 8.6 | -11.2 | 17.2 | 71.9 | 0.0 |
| Oct | 22.7 | 6 | 97.1 | -91.1 | -336.1 | 3.5 | -5.2 | 11.2 | 85.9 | 0.0 |
| Nov | 22.9 | 12 | 96.8 | -84.8 | -420.9 | 1.5 | -2.0 | 14.0 | 82.8 | 0.0 |
| Dec | 22.6 | 47 | 97.2 | -50.2 | -471.1 | 0.9 | -0.6 | 47.6 | 49.6 | 0.0 |
| Ann | 21.8 | 1,205 | 1,029.2 | 175.8 |  | 580.2 | 0.0 | 657.2 | 372.0 | 547.8 |
|  |  |  |  |  |  |  |  |  |  |  |
| *Site 8 – Ubajara, CE* | | | | | | | | | | |
| Jan | 22.3 | 139 | 94.3 | 44.7 | -77.6 | 46.0 | 44.7 | 94.3 | 0.0 | 0.0 |
| Feb | 21.7 | 227 | 79.1 | 147.9 | 0.0 | 100.0 | 54.0 | 79.1 | 0.0 | 93.9 |
| Mar | 20.7 | 330 | 77.4 | 252.6 | 0.0 | 100.0 | 0.0 | 77.4 | 0.0 | 252.6 |
| Apr | 20.8 | 316 | 75.1 | 240.9 | 0.0 | 100.0 | 0.0 | 75.1 | 0.0 | 240.9 |
| May | 20.7 | 190 | 76.3 | 113.7 | 0.0 | 100.0 | 0.0 | 76.3 | 0.0 | 113.7 |
| Jun | 20.9 | 65 | 75.2 | -10.2 | -10.2 | 90.3 | -9.7 | 74.7 | 0.5 | 0.0 |
| Jul | 21.1 | 30 | 79.7 | -49.7 | -60.0 | 54.9 | -35.4 | 65.4 | 14.4 | 0.0 |
| Aug | 21.6 | 9 | 84.8 | -75.8 | -135.8 | 25.7 | -29.2 | 38.2 | 46.6 | 0.0 |
| Sep | 22.2 | 8 | 88.5 | -80.5 | -216.2 | 11.5 | -14.2 | 22.2 | 66.3 | 0.0 |
| Oct | 22.5 | 7 | 95.4 | -88.4 | -304.7 | 4.8 | -6.8 | 13.8 | 81.7 | 0.0 |
| Nov | 22.7 | 12 | 95.1 | -83.1 | -387.8 | 2.1 | -2.7 | 14.7 | 80.4 | 0.0 |
| Dec | 22.5 | 50 | 96.6 | -46.6 | -434.4 | 1.3 | -0.8 | 50.8 | 45.8 | 0.0 |
| Ann | 21.6 | 1,383 | 1,017.5 | 365.5 |  | 636.6 | 0.0 | 681.9 | 335.7 | 701.1 |
