# Supplementary material for: Assembly rules in a resource gradient: Competition and abiotic filtering determine the structuring of plant communities in stressful environments
Source: PLoS One. 2020 Mar 13;15(3):e0230097. doi: 10.1371/journal.pone.0230097 (PMC7069682; doi:10.1371/journal.pone.0230097)
Supplement: S3 Table — (DOC) [file pone.0230097.s003.doc]

**S3 Table -** Traits used to measure the phenotypic pattern of woody species in different plant formations in the semi-arid region of northeastern Brazil.

| **Functional traits** | **Functional significance** | **Data type (unit)** | **Reference** |
| --- | --- | --- | --- |
| **1. α traits** |  |  |  |
| a. maximum height | competitive vigour, fecundity, and growth time between disturbances | continuous (m) | [1,2,3,4,5] |
| b. mean biomass | space occupation and resource uptake | continuous (Mg) | [1,3,4,5] |
| c. specific leaf area | leaf lifespan, leaf defence, positively correlated with growth rate and mass-based maximum photosynthetic rate | continuous (mm^2^.mg^-1^) | [1,3,4] |
| d. leaf nitrogen concentration | mass-based maximum photosynthetic rate | continuous  (mg.g^-1^) | [1,3] |
| e. leaf phenology | trade-off between plant growth rate and plant protection (‘defences’) or nutrient conservation. | categorical  (deciduous or evergreen) | [1,3] |
| f. dispersule size | dispersion capacity and resource stock | categorical  (small, medium, large, and very large) | [1,3,5] |
| g. potential hydric conductivity | water transport capacity | continuous (kg.m.MPa^-1^s^-1^) | [4,6] |
| **2. β-traits** |  |  |  |
| h. leaf size | leaf energy and water balance | continuous (mm^2^) | [1,2] |
| i. leaf type | trade-off between reduction of water loss and efficiency in light capture | categorical  (simple, compound, and bicompound) | [1,2] |
| j. wood density | mechanical resistance, trade-off between plant growth and stem defences against pathogens, herbivores or physical damage by abiotic factors. | continuous  (g.cm^-3^) | [1,6,7] |
| l. dispersal mode | (re)colonization ability | categorical  (anemochory, autochory, barochory, zoochory, and myrmechory) | [1,5] |

**References:**

1. Cornelissen JHC, Lavorel S, Garnier E, et al. A handbook of protocols for standardized and easy measurement of plant functional traits worldwide. Aust J Bot 2003; 51: 335-380.
2. Spasojevic MJ, Suding KN. Inferring community assembly mechanisms from functional diversity patterns: the importance of multiple assembly processes. J Ecol 2012; 100: 652-661
3. Kraft NJB, Godoy O, Levine JM. Plant functional traits and the multidimensional nature of species coexistence. Proc Natl Acad Sci USA 2015; 112: 797-802.
4. Violle C, Garnier E, Lecoeur J, Roumet C, Podeur C, Blanchard A, et al. Competition, traits and resource depletion in plant communities. Oecologia 2009; 160: 747-755.
5. Westoby M, Falster DS, Moles A, et al. Plant ecological strategies: some leading dimensions of variation between species. Annu Rev Ecol Syst 2002; 33:125-59.
6. Poorter L, McDonald I, Alarcon A, et al. The importance of wood traits and hydraulic conductance for the performance and life history strategies of 42 rainforest tree species. New Phytol 2009; 185: 481 – 492.
7. Ackerly DD, Schwilk DW, Webb CO. Niche evolution and adaptive radiation: Testing the order of trait divergence. Ecology 2006; 87: S50‑S61.
