# Supplementary material for: Assembly rules in a resource gradient: Competition and abiotic filtering determine the structuring of plant communities in stressful environments
Source: PLoS One. 2020 Mar 13;15(3):e0230097. doi: 10.1371/journal.pone.0230097 (PMC7069682; doi:10.1371/journal.pone.0230097)
Supplement: S4 Table — (DOC) [file pone.0230097.s004.doc]

**S4 Table.** Results of the comparisons between real variation represented by individual PCA axes and relevant variation calculated by the broken-stick model.

|  | axis1 | axis2 | axis3 | axis4 | axes5 |
| --- | --- | --- | --- | --- | --- |
| percentage of variance | **45.31%** | **39.21%** | 10.23% | 3.83% | 1.27% |
| cumulative percentage of variance | 45.31% | 84.52% | 94.75% | 98.58% | 99.85% |
| broken-stick percentage | 37.04% | 22.75% | 15.61% | 10.85% | 7.28% |
| broken-stick cumulative | 37.04% | 59.79% | 75.40% | 86.25% | 93.53% |
|  |  |  |  |  |  |
