# Supplementary material for: Assembly rules in a resource gradient: Competition and abiotic filtering determine the structuring of plant communities in stressful environments
Source: PLoS One. 2020 Mar 13;15(3):e0230097. doi: 10.1371/journal.pone.0230097 (PMC7069682; doi:10.1371/journal.pone.0230097)
Supplement: S1 Fig — (DOC) [file pone.0230097.s005.doc]

**Histograms with the distributions of null model values for each metric analysed**


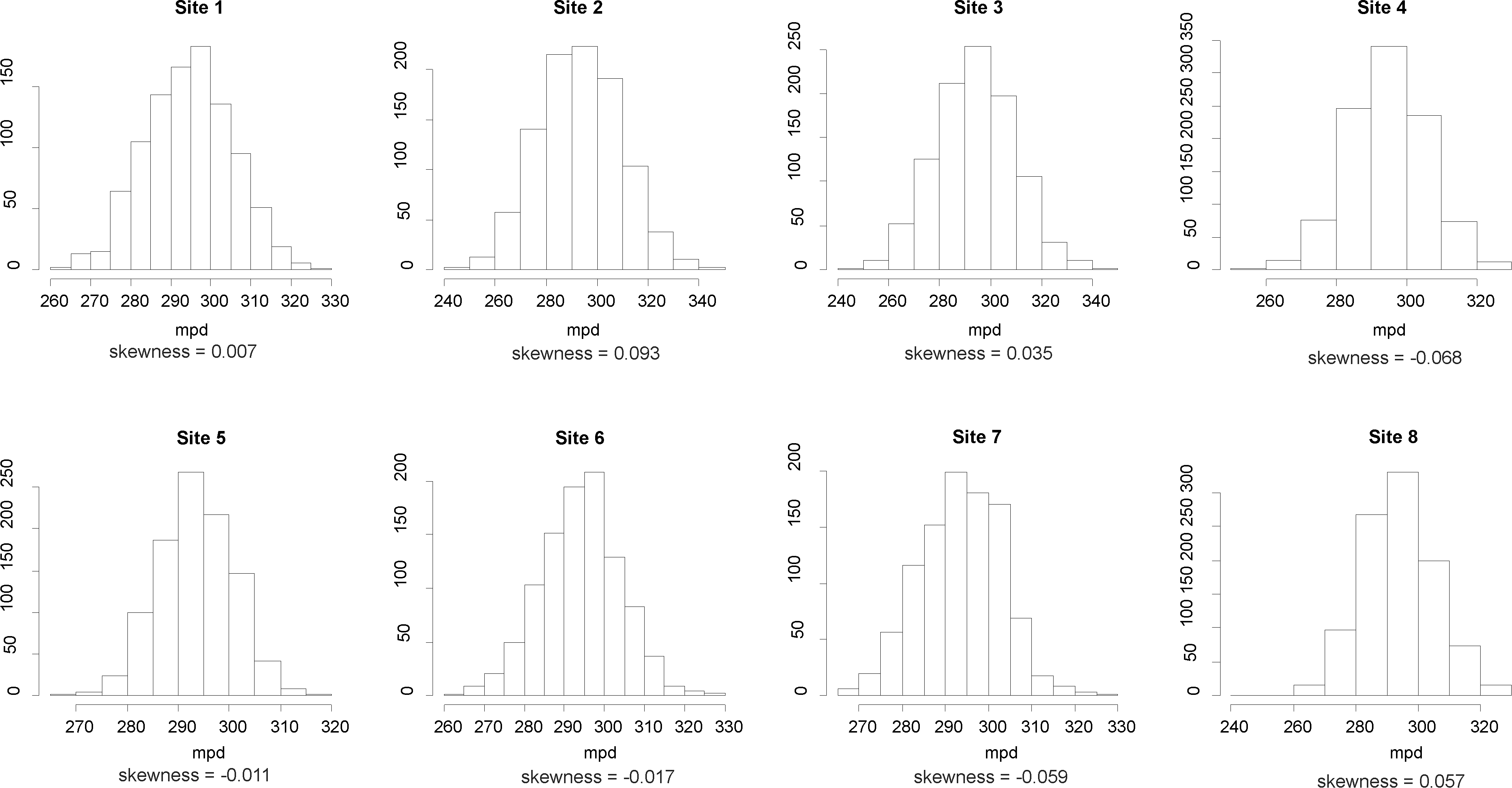


**S1-A Fig.** Distributions of null model values for MPD


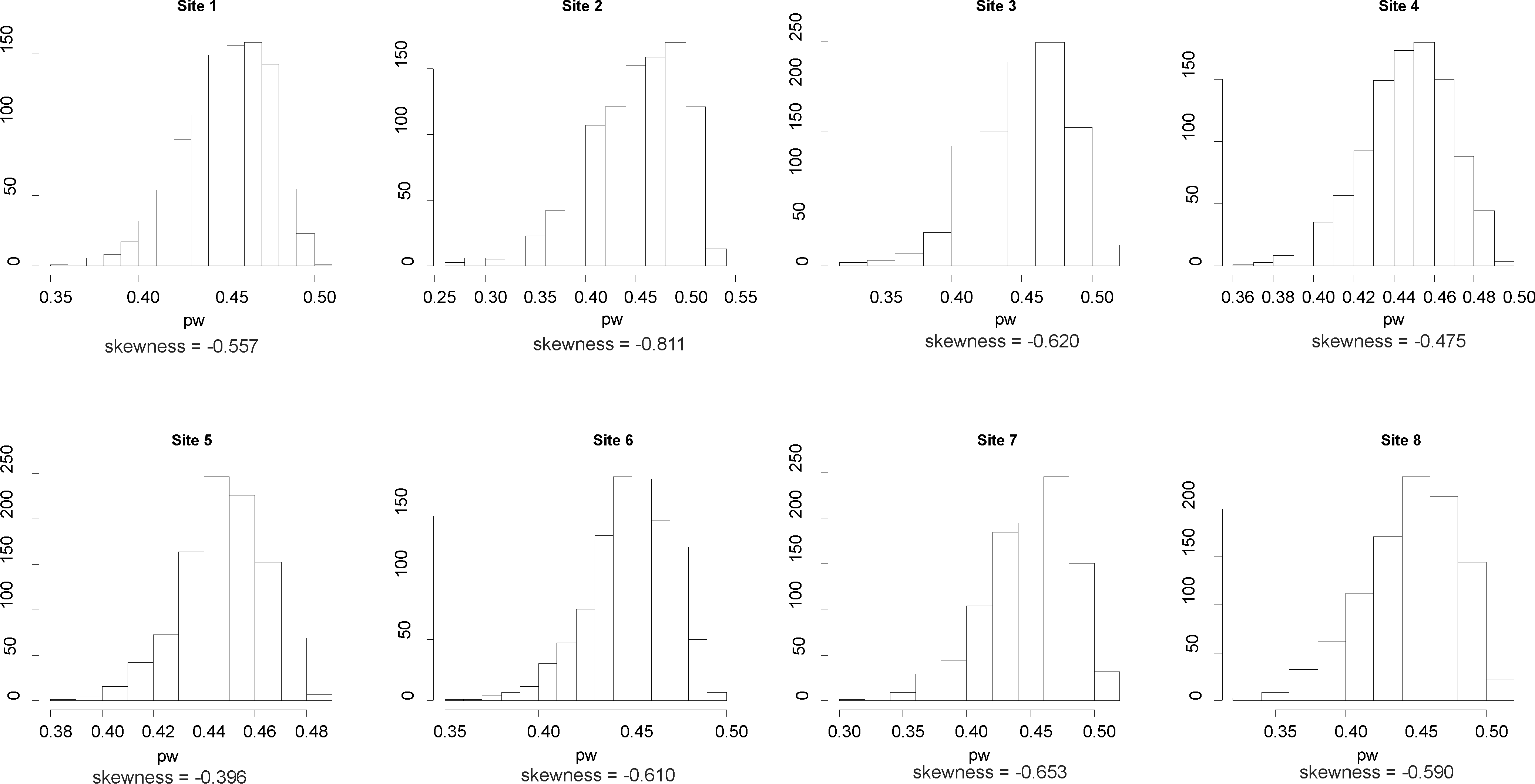


**S1-B Fig.** Distributions of null model values for PW


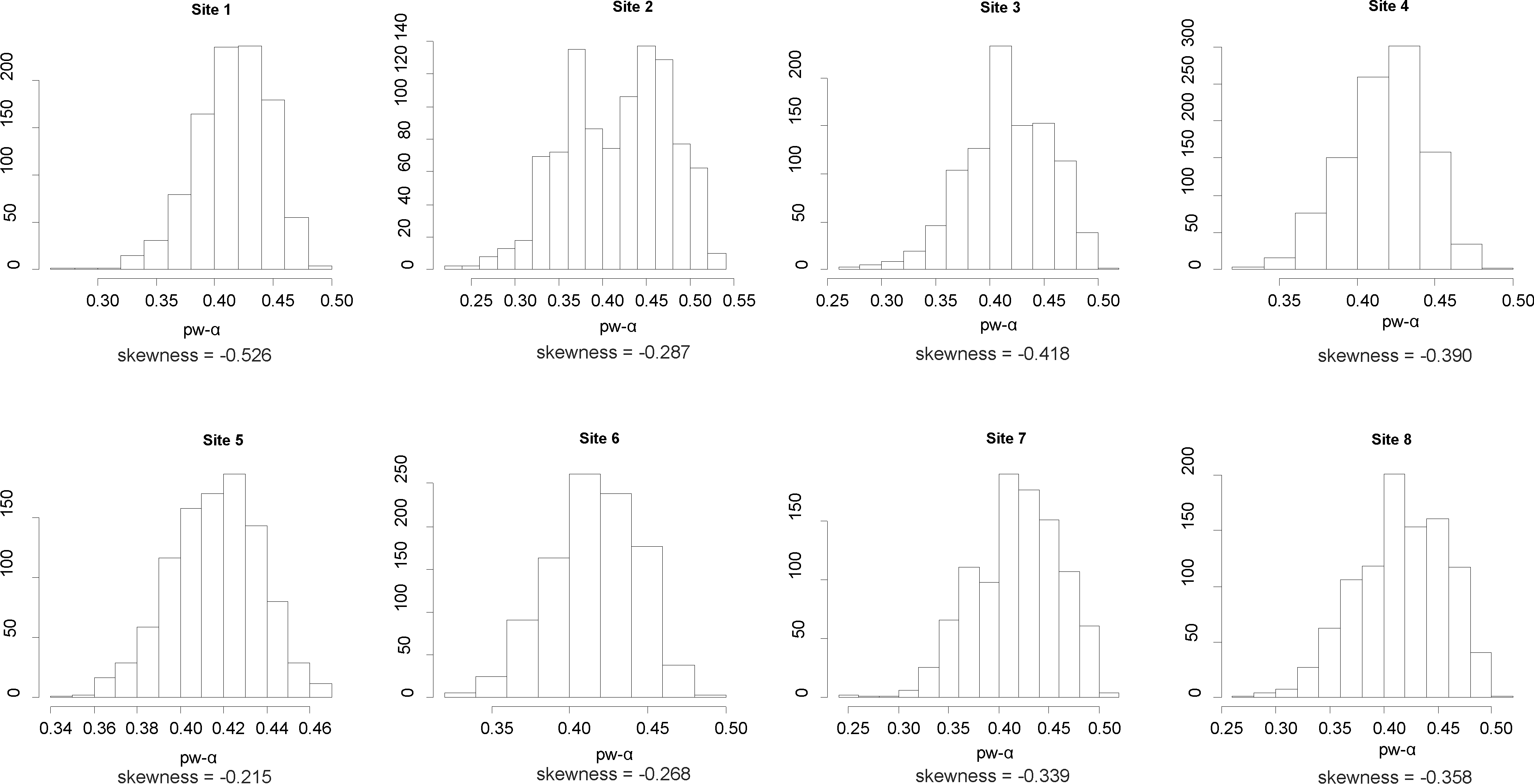


**S1-C Fig.** Distributions of null model values for PW-α


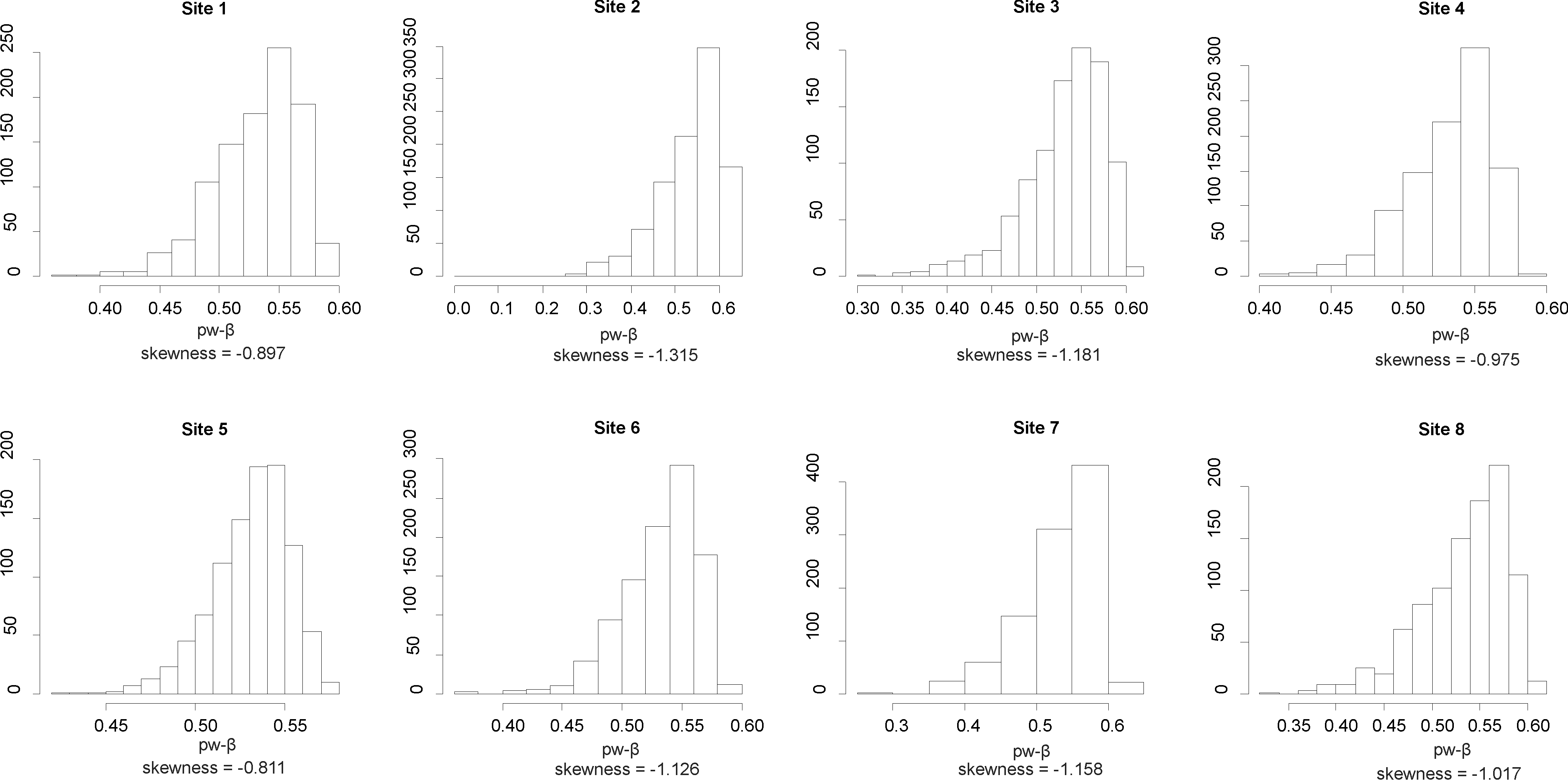


**S1-D Fig.** Distributions of null model values for PW-β
