# Supplementary material for: Assembly rules in a resource gradient: Competition and abiotic filtering determine the structuring of plant communities in stressful environments
Source: PLoS One. 2020 Mar 13;15(3):e0230097. doi: 10.1371/journal.pone.0230097 (PMC7069682; doi:10.1371/journal.pone.0230097)
Supplement: S1 Text — (DOC) [file pone.0230097.s006.doc]

**S1 - Text. Phylobetadiversity analyses**

We calculated phylobetadiversity through two indices: ses.comdist and Sorenson index of phylobetadiversity. The former has the advantage of enabling the comparison between observed data and a null distribution, checking how much the results differ from expected at random. The latter (more frequently used in community studies) has the advantage that it can be decomposed and, therefore, we can check the specific effect of the species turnover between communities. Ses.comdist is a measure of phylogenetic distance between two communities [1]. It is a metric analogous to ses.mpd, in which the average phylogenetic distance between pairs of species of two communities is calculated. This distance is compared to a null model formed by re-sampling of the species pool. In our study, we used the same algorithm used for ses.mpd: phylogeny.pool. The interpretation of the results of ses.comdist is similar to that of ses.mpd. Negative values indicate phylogenetic aggregation, i.e., species from two communities are phylogenetically closer than expected at random and positive values indicate the opposite: phylogenetic dispersion. The Sorenson index of phylobetadiversity measures the gain or loss (nestedness) and substitution (turnover) of species between two communities [2]. The nestedness reflects whether the species composition of a community is a subset of another community. Hence, it is possible to indicate whether a decomposition of the regional pool causes a decrease in number of species of a given community. On the other hand, turnover indicates whether the set of species of one community is being replaced with another set of species. In our case, a high turnover between communities could indicate that there are distinct regional pools for different communities.

We found that, although the sites are geographically distant (ca. 320 km), there was high phylogenetic similarity among them. There was phylogenetic aggregation among five of the eight sites analysed, which indicates that the species of these sites were more closely related to one another than expected at random (Table A). Only the comparisons involving the most humid sites (S7 and S8) showed positive phylobetadiversity values. Among possible combinations involving the eight sites, only three (11%) showed a dispersed pattern for phylobetadiversity. Hence, we can conclude that despite the impoverishment in the number of taxa as water restriction increases, there were no significant changes regarding the representativeness of the main clades. We also found that geographic distance had low influence on phylogenetic turnover (Fig A). Therefore, it is possible that there is a single regional pool for all communities.

**Table A**. Phylobetadiversity (ses.comdist) among sites

| Sites | S1 | S2 | S3 | S4 | S5 | S6 | S7 |
| --- | --- | --- | --- | --- | --- | --- | --- |
| S2 | **-3.487** |  |  |  |  |  |  |
| S3 | **-2.457** | **-2.112** |  |  |  |  |  |
| S4 | **-2.166** | **-2.061** | -1.466 |  |  |  |  |
| S5 | **-2.128** | -1.636 | -1.137 | **-1.962** |  |  |  |
| S6 | -0.646 | -0.125 | -0.971 | -0.379 | -0.263 |  |  |
| S7 | 1.724 | 1.435 | 1.014 | 1.617 | **2.410** | **2.109** |  |
| S8 | 1.457 | 1.231 | 0.891 | 1.313 | 1.814 | 1.733 | **2.886** |

Significant results are shown in **bold**

**
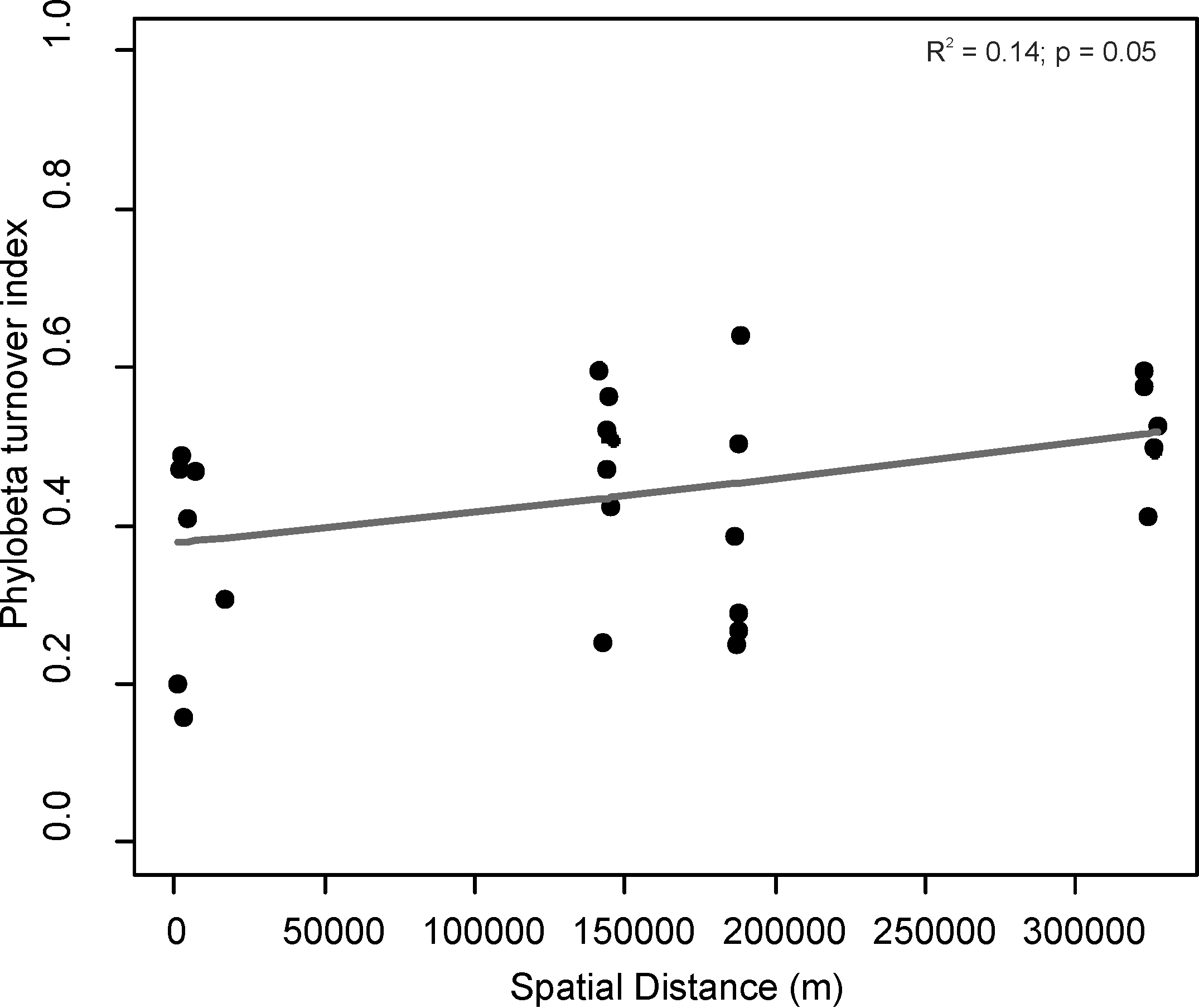
**

**Fig. A.** Correlation between phylobeta turnover index and the spatial distance between pairs of plots

**References:**

1. Webb CO, Ackerly DD, Kembel SW. Phylocom: software for the analysis of community phylogenetic structure and trait evolution. Bioinformatics 2008; 24: 2098-2100.

2. Baselga, A. Partitioning the turnover and nestedness components of beta diversity. Glob. Ecol. Biogeogr. 2010; 19: 134-143.
